# Supplementary figures and images for: Linking skeletal muscle aging with osteoporosis by lamin A/C deficiency
Source: PLoS Biol. 2020 Jun 1;18(6):e3000731. doi: 10.1371/journal.pbio.3000731 (PMC7310860; doi:10.1371/journal.pbio.3000731)

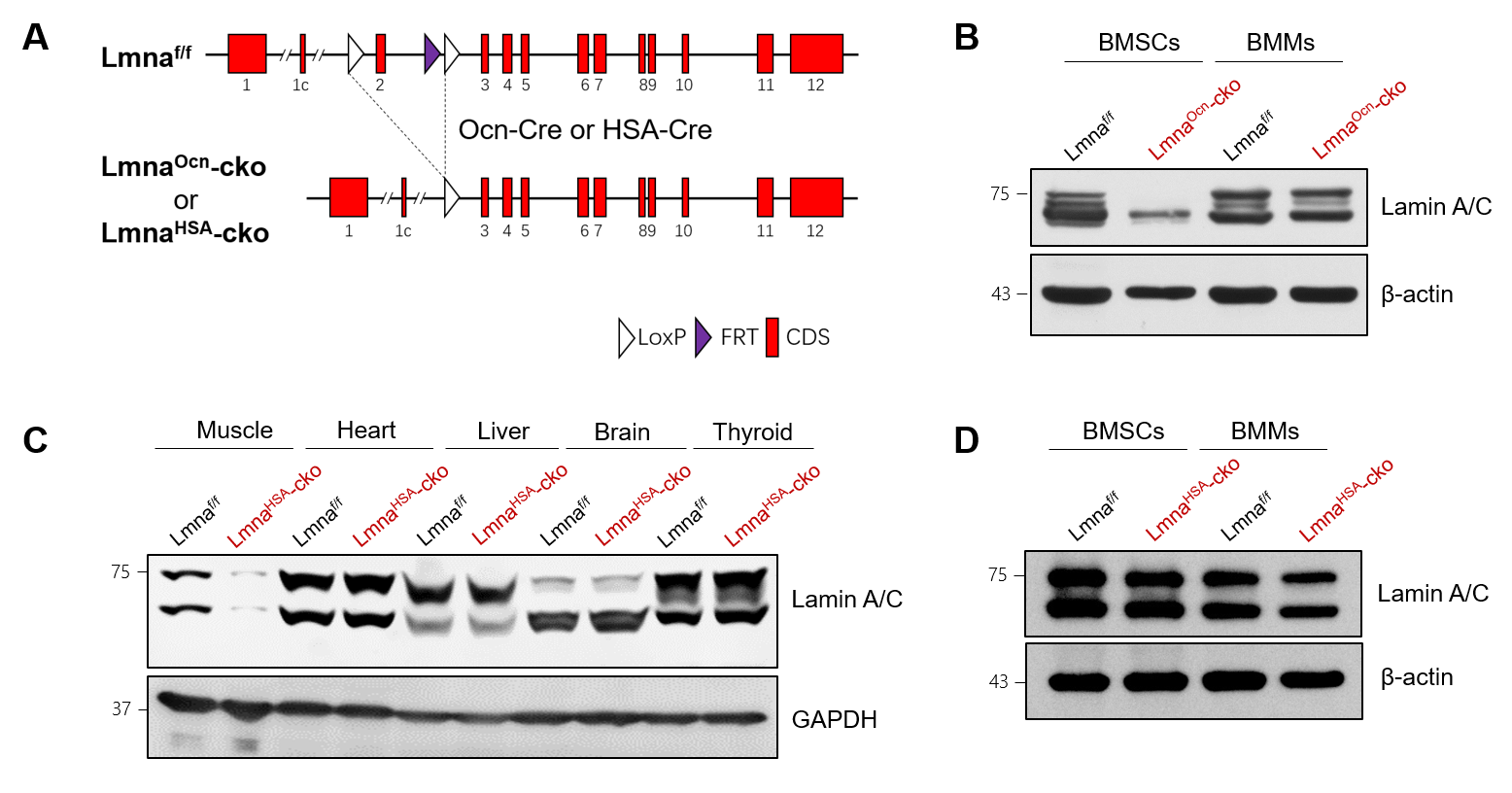

Supplement: S1 Fig — (A) Strategy to cleave exon 2 of Lmna flanked by loxP sites in the Lmnaflox allele. Lmnaflox/flox mice were crossed with Ocn-Cre or the HSA-Cre transgenic mice to generate OB- and muscle-selective cko mutant mice, LmnaOcn-cko and LmnaHSA-cko. (B) Western blotting analysis of lamin A/C expression in primary cultured BMSCs and BMMs from 3-mo Lmnaf/f and LmnaOcn-cko mice. β-Actin was used as the loading control. (C) Western blotting analysis of lamin A/C expression in different tissue of 3-mo Lmnaf/f and LmnaHSA-cko mice. GAPDH was used as the loading control. (D) Western blotting analysis of lamin A/C expression in primary cultured BMSCs and BMMs from 3-mo Lmnaf/f and LmnaHSA-cko mice. β-Actin was used as the loading control. BMM, bone marrow macrophage/monocyte; BMSC, bone marrow stromal cell; cko, conditional knockout; Cre, cyclization recombination enzyme; HSA, human alpha-skeletal actin; KO, knockout; Lmna, lamin A/C gene; Lmnaf/f, floxed Lmna mice; LmnaHSA-cko, skeletal muscle–specific Lmna-cko mice; LmnaOcn-cko, OB-selective Lmna–conditional knockout mice; mo, months old; OB, osteoblast; Ocn, osteocalcin. (TIF) [file pbio.3000731.s001.tif]

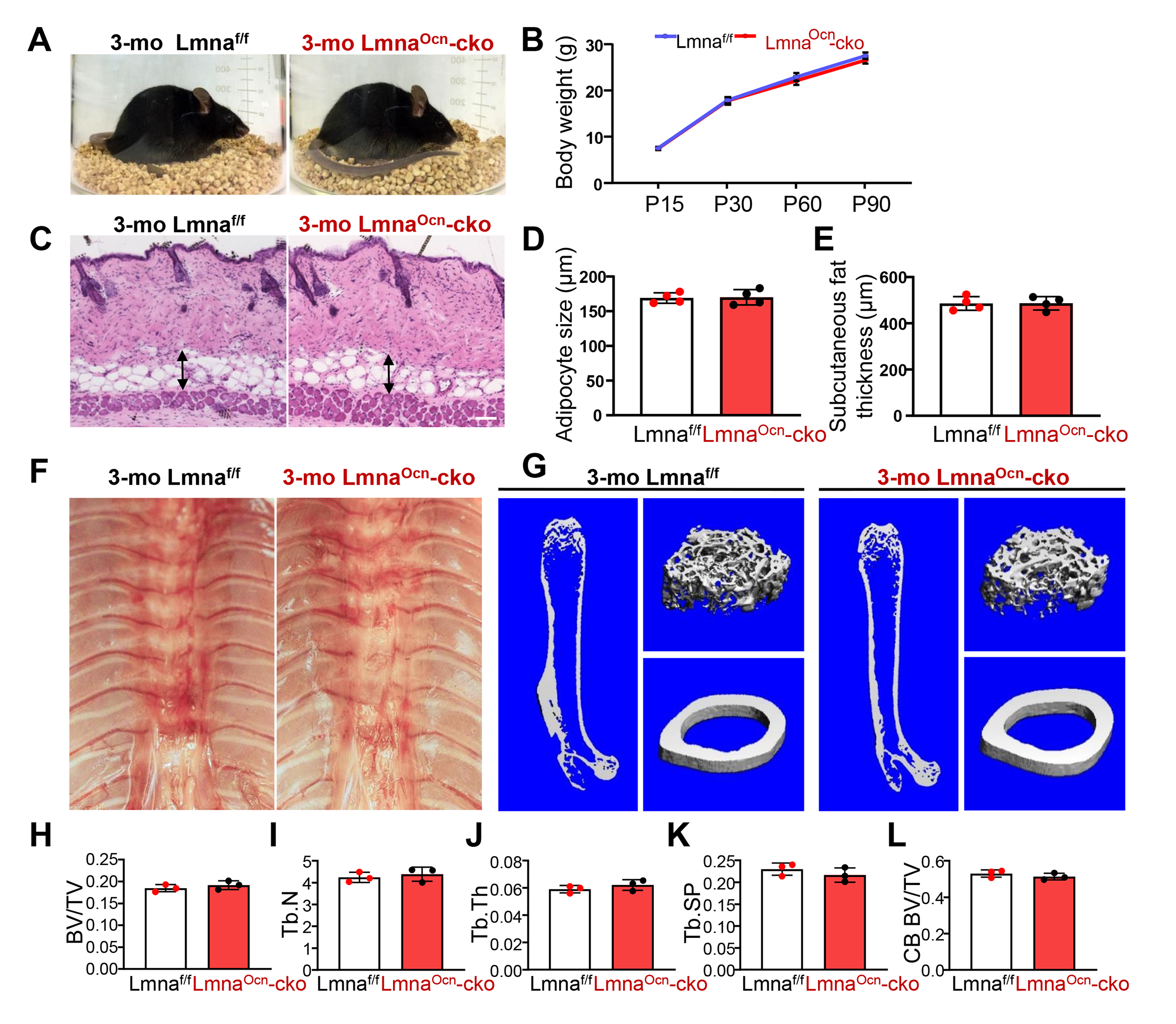

Supplement: S2 Fig — (A) Photo of a 3-mo LmnaOcn-cko mouse and a littermate Lmnaf/f mouse. (B) No change of body weight in the LmnaOcn-cko mouse. (C) Representative HE-stained sections of skin from 3-mo Lmnaf/f and LmnaOcn-cko mice. Scale bar, 200 μm. (D,E) Quantification analyses of adipocyte size and subcutaneous fat thickness of 3-mo Lmnaf/f and LmnaOcn-cko mice. N = 4. (F) Thorax of 3-mo Lmnaf/f and LmnaOcn-cko mice. (G) The μCT analysis of femurs from 3-mo Lmnaf/f and LmnaOcn-cko littermates. Three different male mice of each genotype per group were examined blindly. (H-L) Quantification analyses (n = 3) of TB BV/TV, Tb.N, Tb.Th, Tb.Sp, and CB BV/TV by the direct model of μCT analysis. The underlying data for this figure can be found in S1 Data. μCT, microcomputer tomographic; BV/TV, bone volume over total volume; CB, cortical bone; cko, conditional knockout; Lmna, lamin A/C gene; Lmnaf/f, floxed Lmna mice; LmnaOcn-cko, OB-selective Lmna–conditional knockout mice; mo, months old; OB, osteoblast; Ocn, osteocalcin; TB, trabecular bone; Tb.N, trabecular bone number; Tb.Sp, trabecular bone space; Tb.Th, trabecular bone thickness. (TIF) [file pbio.3000731.s002.tif]

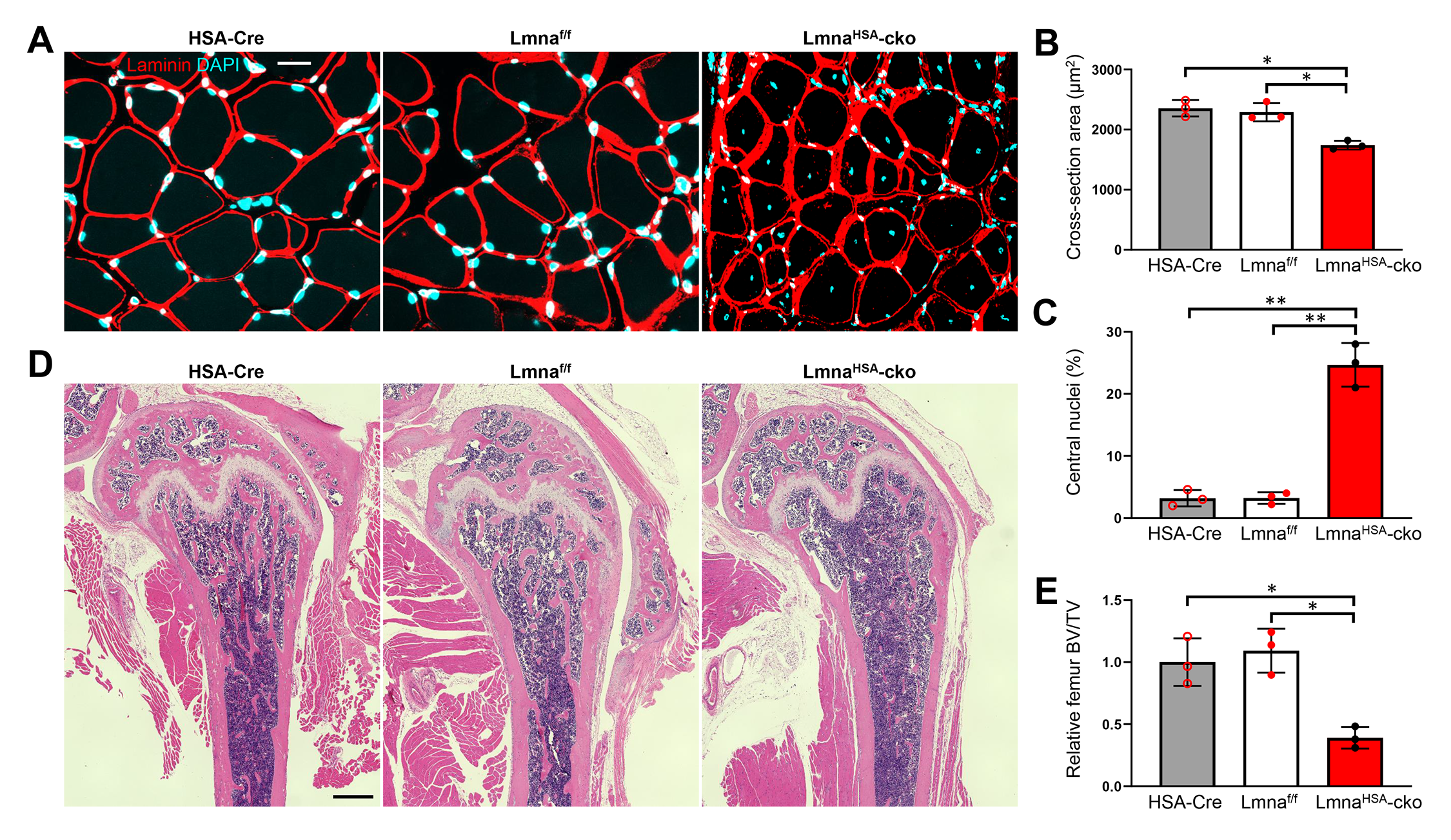

Supplement: S3 Fig — (A) Representative images of gastrocnemius cross sections from 3-mo HSA-Cre, Lmnaf/f, and LmnaHSA-cko mice. Scale bar, 20 μm. (B,C) Quantification analyses of cross-section area and central nuclei distribution. N = 3 mice per group. *P < 0.05, **P < 0.01. (D) Histomorphological examinations of the femur from 3-mo HSA-Cre, Lmnaf/f, and LmnaHSA-cko mice by HE staining analysis. Scale bar, 300 μm. (E) Quantification analysis of data from (D). N = 3 mice, *P < 0.05. The underlying data for this figure can be found in S1 Data. cko, conditional knockout; Cre, cyclization recombination enzyme; HSA, human alpha-skeletal actin; Lmna, lamin A/C gene; Lmnaf/f, floxed Lmna mice; LmnaHSA-cko, skeletal muscle–specific Lmna-cko mice; mo, months old; Ocn, osteocalcin. (TIF) [file pbio.3000731.s003.tif]

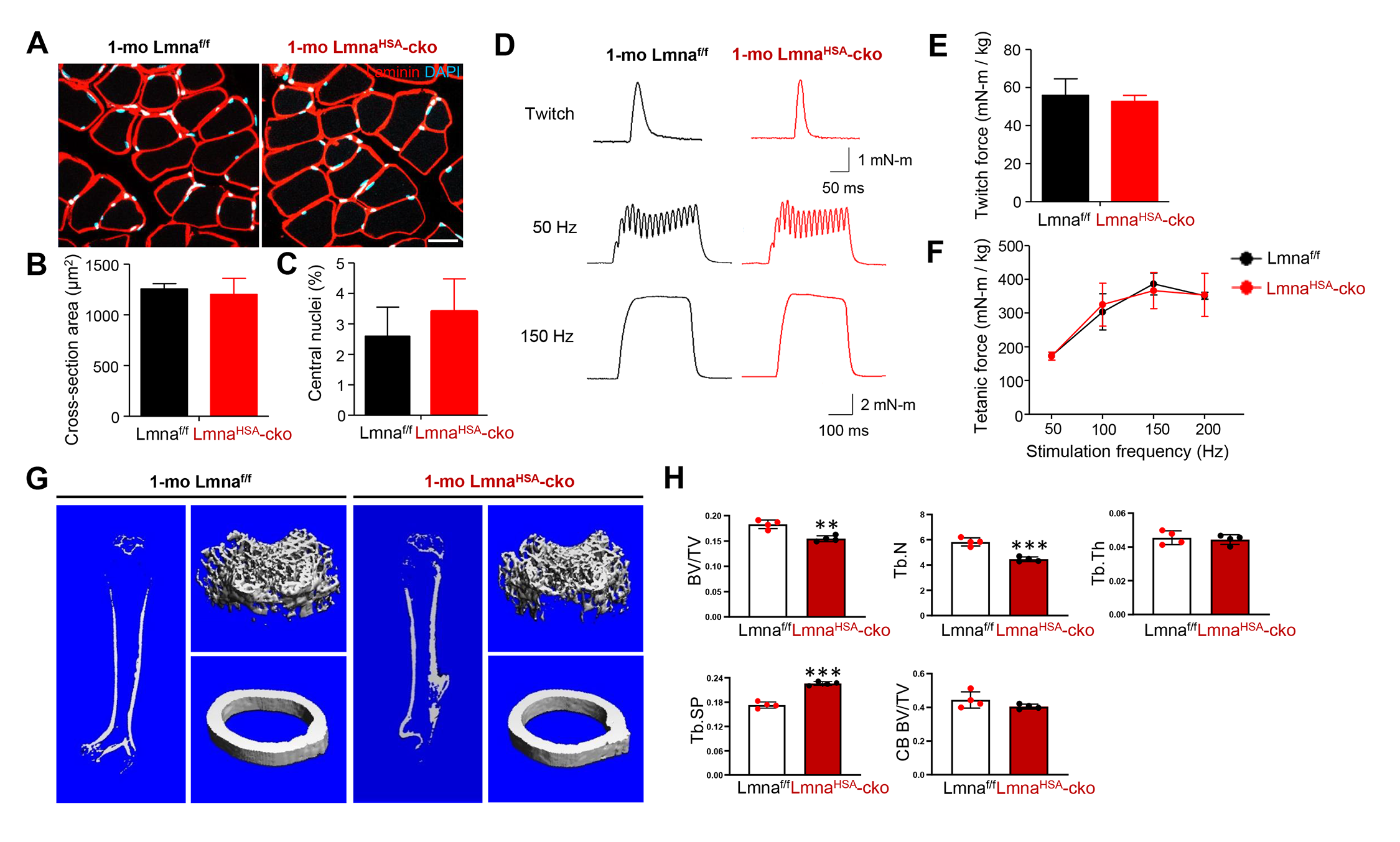

Supplement: S4 Fig — (A) Representative images of gastrocnemius cross sections. Scale bar, 20 μm. (B,C) Quantification analyses of cross-section area and central nuclei distribution. N = 3 mice per group. (D) Representative twitch curves and tetanic curves at stimulation frequencies 50 and 150 Hz by muscle stimulation. (E,F) Quantification analyses of twitch force and tetanic force. N = 4 mice per group. (G) The μCT analysis of femurs from 1-mo Lmnaf/f and LmnaHSA-cko littermates. Four different male mice of each genotype per group were examined blindly. (H) Quantification analyses (n = 4) of TB BV/TV, Tb.N, Tb.Th, Tb.Sp, and CB BV/TV by the direct model of μCT analysis. Data is determined by two-way ANOVA. **P < 0.01, ***P < 0.001, significant difference. The underlying data for this figure can be found in S1 Data. μCT, microcomputer tomographic; BV/TV, bone volume over total volume; CB, cortical bone; cko, conditional knockout; HSA, human alpha-skeletal actin; Lmna, lamin A/C gene; Lmnaf/f, floxed Lmna mice; LmnaHSA-cko, skeletal muscle–specific Lmna-cko mice; mo, months old; TB, trabecular bone; Tb.N, trabecular bone number; Tb.Sp, trabecular bone space; Tb.Th, trabecular bone thickness. (TIF) [file pbio.3000731.s004.tif]

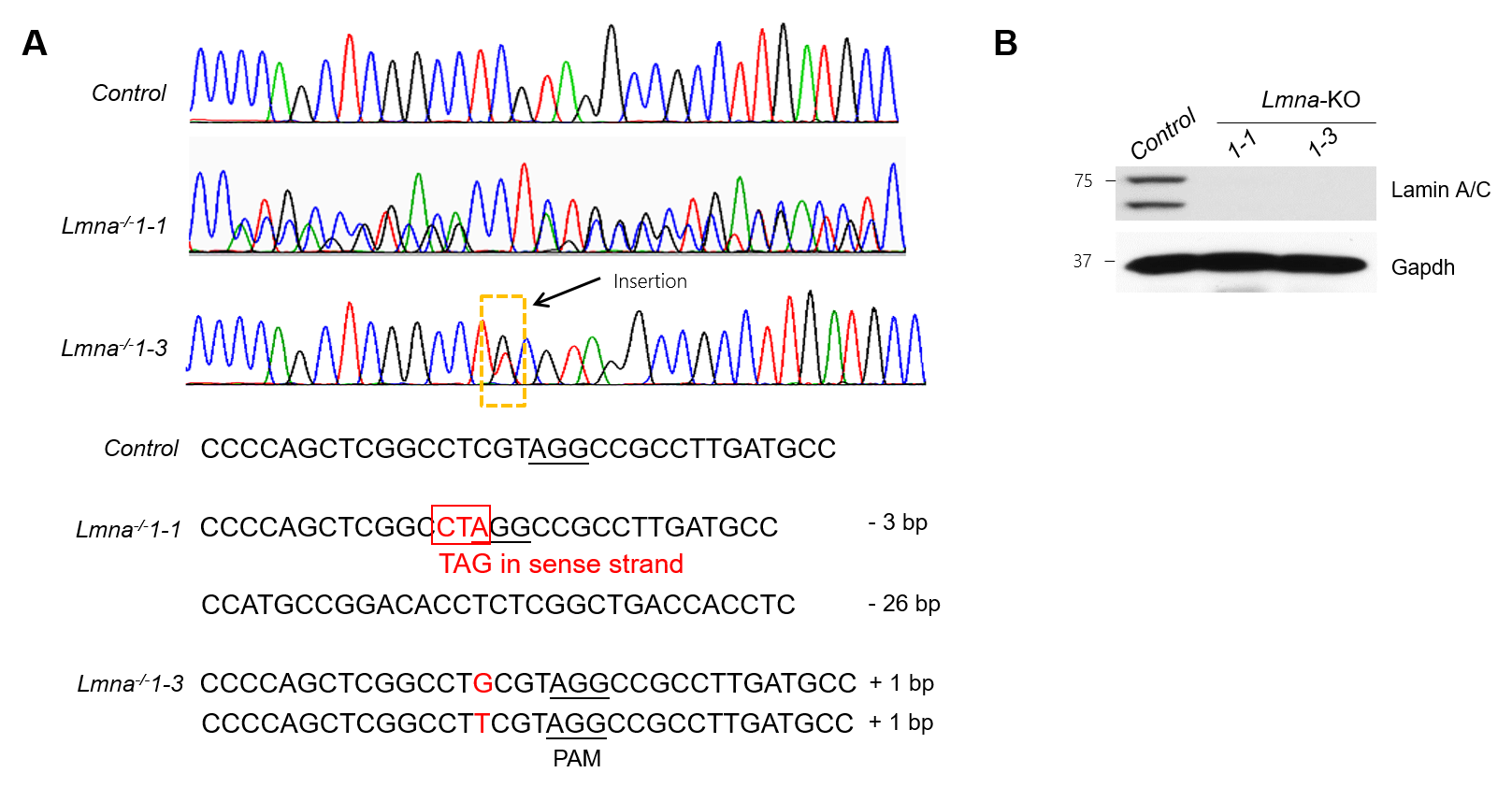

Supplement: S5 Fig — (A) Sequencing data showing the frameshift or/and terminal codon generated by NHEJ in Lmna-KO cell lines 1–1 and 1–3. (B) Western blot showing KO of lamin A/C in C2C12 cell lines. cko, conditional knockout; KO, knockout; Lmna, lamin A/C gene; NHEJ, nonhomologous end joining. (TIF) [file pbio.3000731.s005.tif]

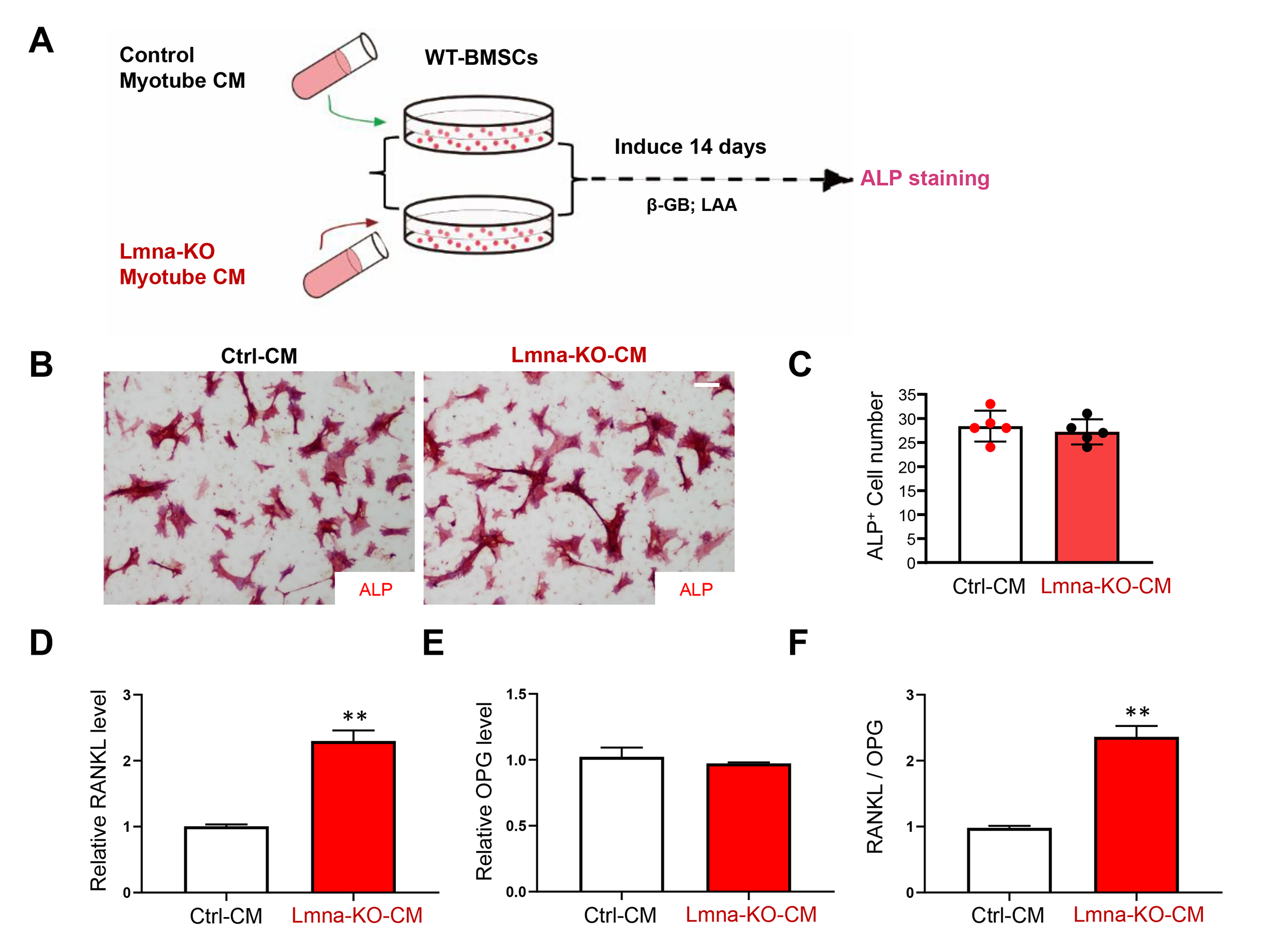

Supplement: S6 Fig — (A) Experimental strategy. (B) ALP staining analysis of cultured BMSCs in the presence of CM of Ctrl or Lmna-KO myotube CM. Scale bar, 100 μm. (C) Quantitative data of the average ALP activities (mean ± SD from five different cultures). (D-F) Real-time PCR analysis of RANKL and OPG expression in OBs treated with CMs of Ctrl or Lmna-KO myotubes. **P < 0.01, significant difference from the Ctrl. The underlying data for this figure can be found in S1 Data. ALP, alkaline phosphatase; BMSC, bone marrow stromal cell; CM, conditioned medium; Ctrl, control; KO, knockout; Lmna, lamin A/C gene; OB, osteoblast; OPG, osteoprotegerin; RANKL, receptor activator of nuclear factor κB ligand. (TIF) [file pbio.3000731.s006.tif]

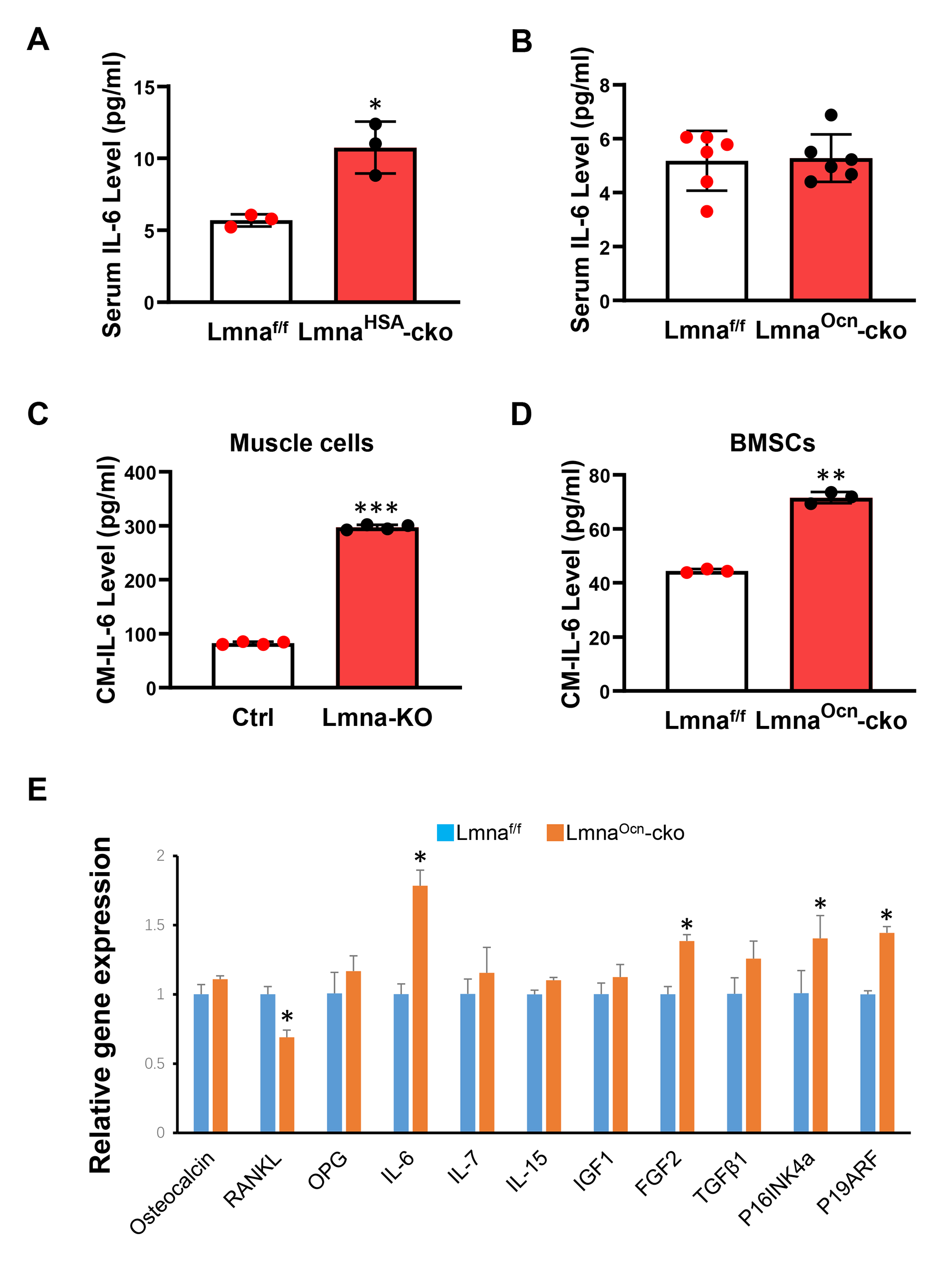

Supplement: S7 Fig — (A) ELISA analysis of serum IL-6 level in 3-mo Lmnaf/f and LmnaHSA-cko mice. *P < 0.05, significant difference. (B) Serum IL-6 level in 3-mo Lmnaf/f and LmnaOcn-cko mice. (C) ELISA analysis of IL-6 level in the culture medium of ctrl and Lmna-KO myotubes induced from C2C12. ***P < 0.001, significant difference. (D) IL-6 levels in the culture medium of BMSCs derived from 3-mo Lmnaf/f and LmnaOcn-cko mice. **P < 0.01, significant difference. (E) Real-time PCR analysis of gene expression in BMSCs derived from 3-mo Lmnaf/f and LmnaOcn-cko mice. *P < 0.05, significant difference. The underlying data for this figure can be found in S1 Data. BMSC, bone marrow stromal cell; cko, conditional knockout; ctrl, control; HSA, human alpha-skeletal actin; IL, interleukin; KO, knockout; Lmna, lamin A/C gene; Lmnaf/f, floxed Lmna mice; LmnaHSA-cko, skeletal muscle–specific Lmna-cko mice; LmnaOcn-cko, OB-selective Lmna–conditional knockout mice; mo, months old; OB, osteoblast; Ocn, osteocalcin. (TIF) [file pbio.3000731.s007.tif]

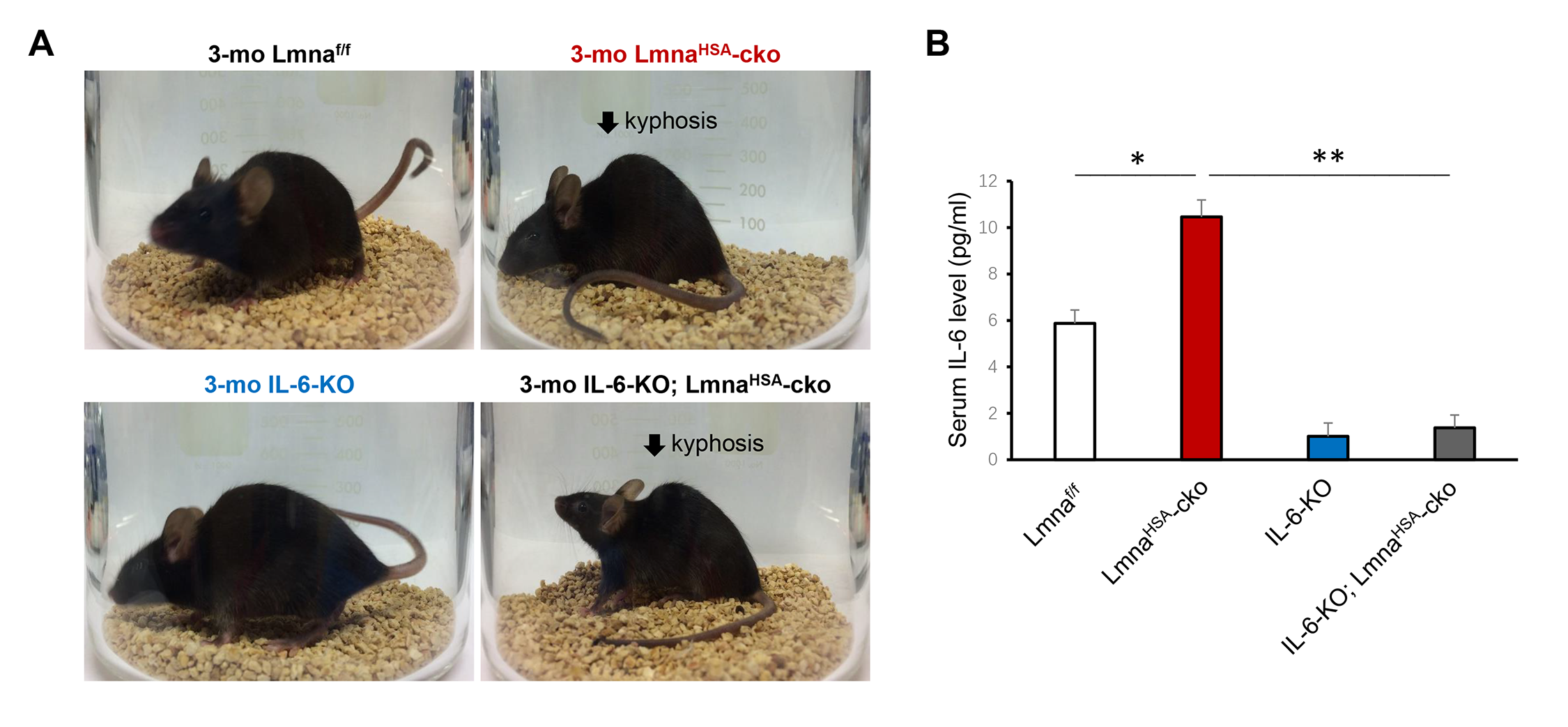

Supplement: S8 Fig — (A) Mouse images of indicated genotypes at 3 mo. The LmnaHSA-cko and IL-6–KO; LmnaHSA-cko mice showed kyphotic phenotype. (B) ELISA analysis of serum levels of IL-6 in 3-mo mice. *P < 0.05, **P < 0.01, significant difference. The underlying data for this figure can be found in S1 Data. cko, conditional knockout; HSA, human alpha-skeletal actin; IL, interleukin; KO, knockout; Lmna, lamin A/C gene; LmnaHSA-cko, skeletal muscle–specific Lmna-cko mice; mo, months old. (TIF) [file pbio.3000731.s008.tif]

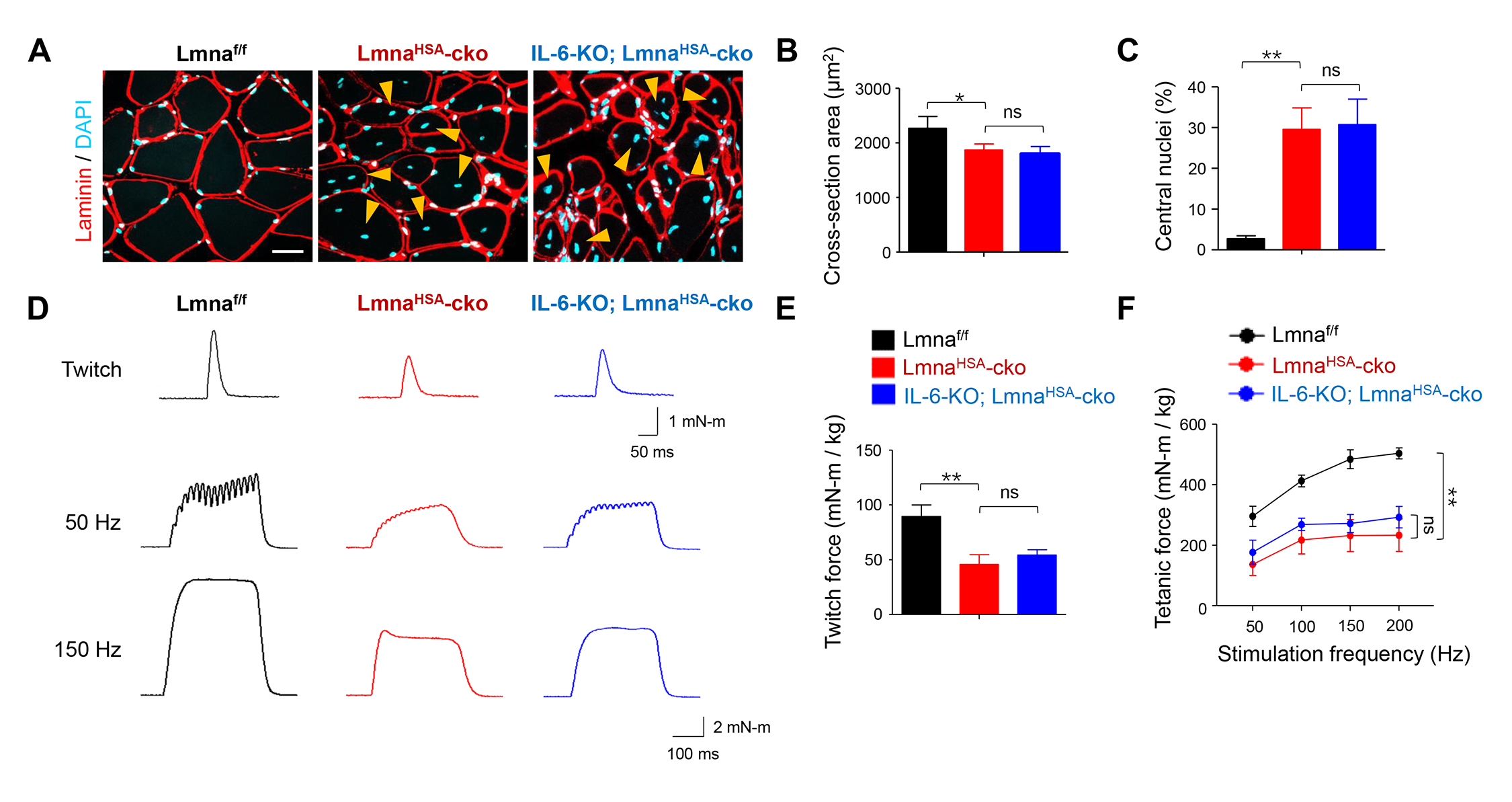

Supplement: S9 Fig — (A) Representative images of gastrocnemius cross sections. Scale bar, 20 μm. (B,C) Quantification analyses of cross-section area and central nuclei distribution. N = 5 mice per group. *P < 0.05, **P < 0.01. (D) Representative twitch curves and tetanic curves at stimulation frequencies 50 and 150 Hz by muscle stimulation. (E,F) Quantification analyses of twitch force and tetanic force. **P < 0.01. N = 4 mice per group. The underlying data for this figure can be found in S1 Data. cko, conditional knockout; HSA, human alpha-skeletal actin; IL, interleukin; Lmna, lamin A/C gene; LmnaHSA-cko, skeletal muscle–specific Lmna-cko mice. (TIF) [file pbio.3000731.s009.tif]

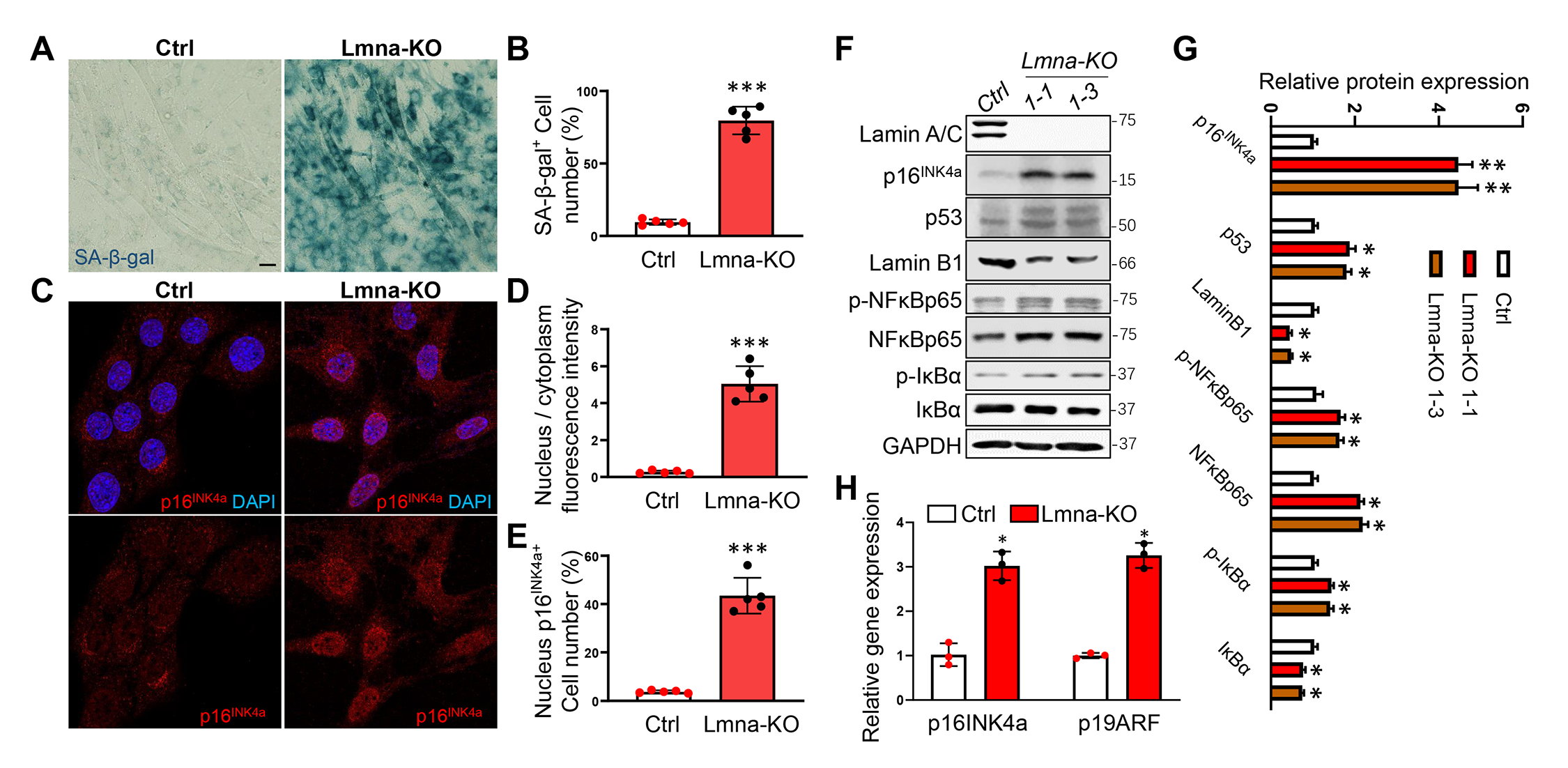

Supplement: S10 Fig — (A) Representative images of SA-β-gal staining of ctrl and Lmna-KO muscle cells. Scale bar, 20 μm. (B) Quantification of SA-β-gal+ cells. ***P < 0.001. (C) Immunostaining analysis of p16INK4a in ctrl and Lmna-KO C2C12 cells. (D,E) Quantification analysis (means ± SD, n = 20 cells from five different assays, ***P < 0.001). (F) Western blot analysis of indicated protein expression in ctrl and Lmna-KO muscle cells. GAPDH was used as the loading ctrls. (G) Quantification analysis (mean ± SD; n = 3). *P < 0.05, ** P < 0.01. (H) Real-time PCR analysis of p16INK4a and p19ARF expression in ctrl and Lmna-KO muscle cells. *P < 0.05, significant difference. The underlying data for this figure can be found in S1 Data. ctrl, control; KO, knockout; Lmna, lamin A/C gene; p19ARF, the alternate reading frame tumor-suppressor protein; SA-β-gal, senescence-associated beta-galactosidase. (TIF) [file pbio.3000731.s010.tif]

Fig 5A

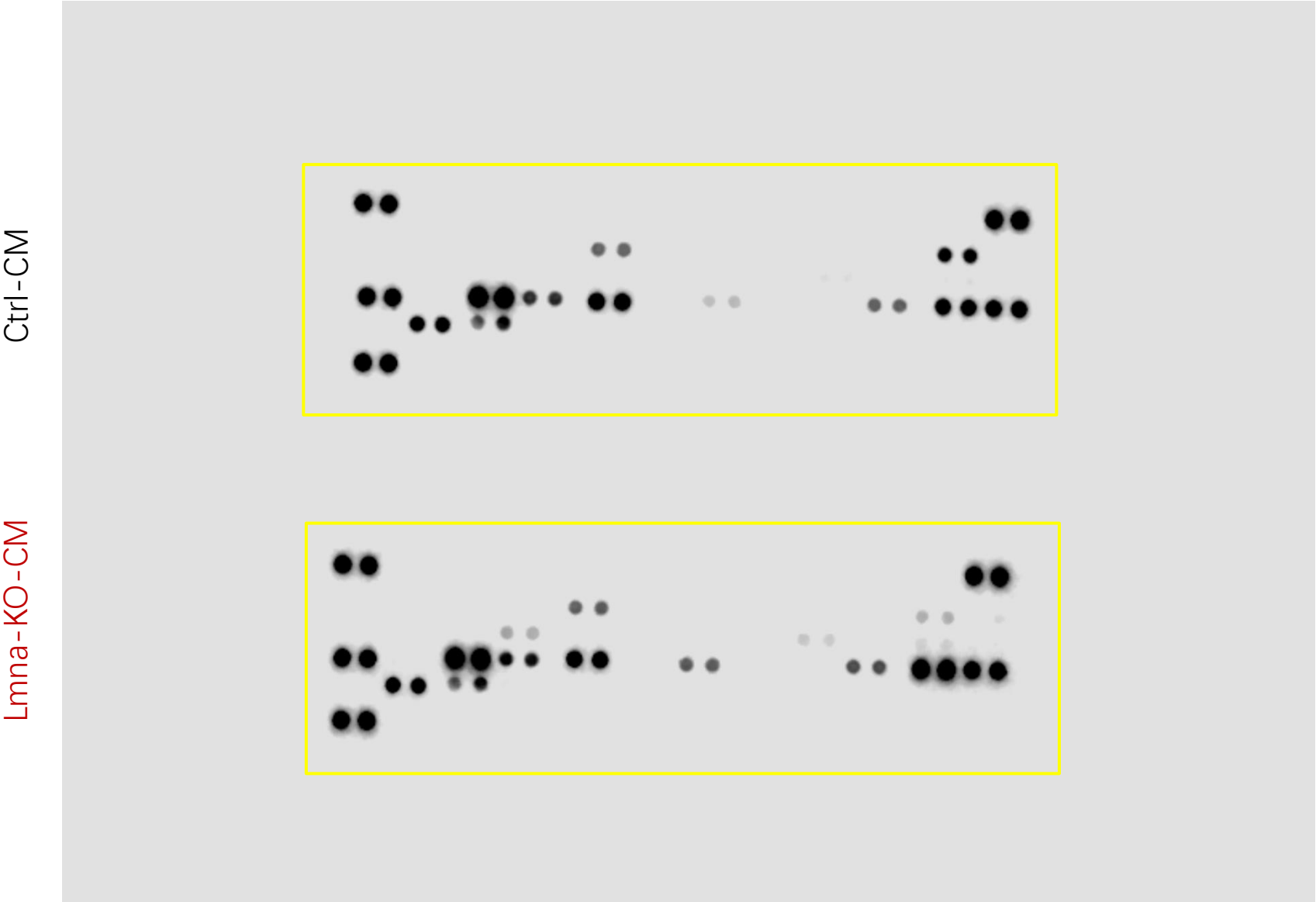

Fig 5D

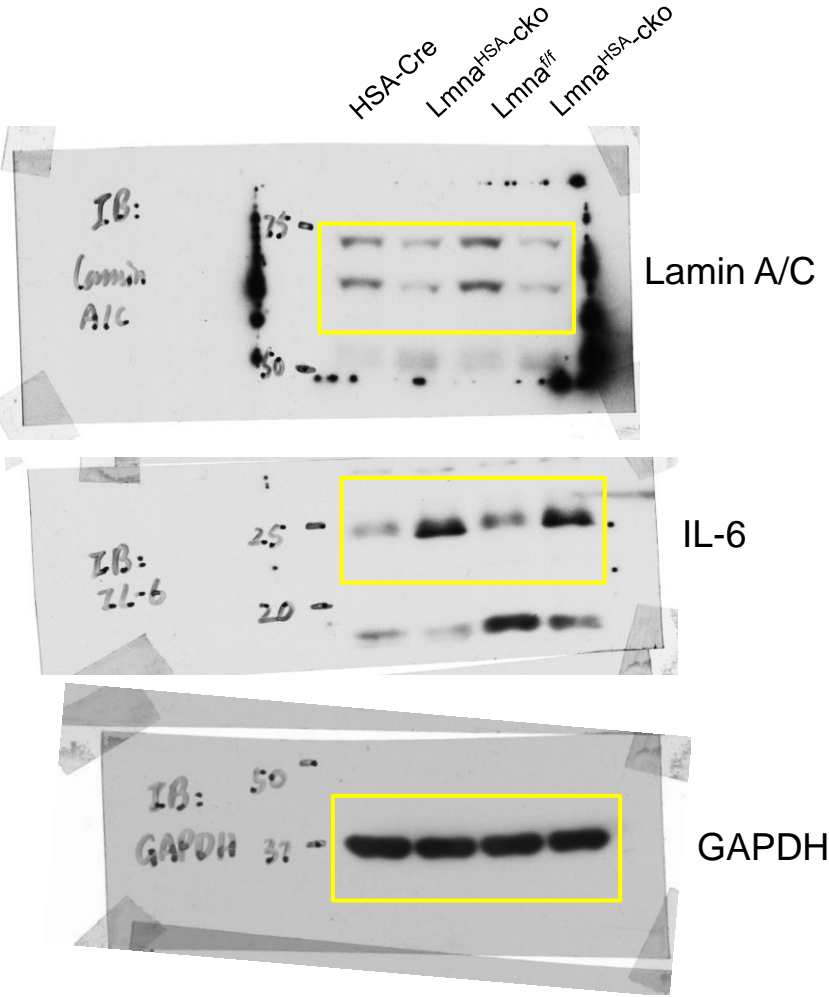

Fig 8C

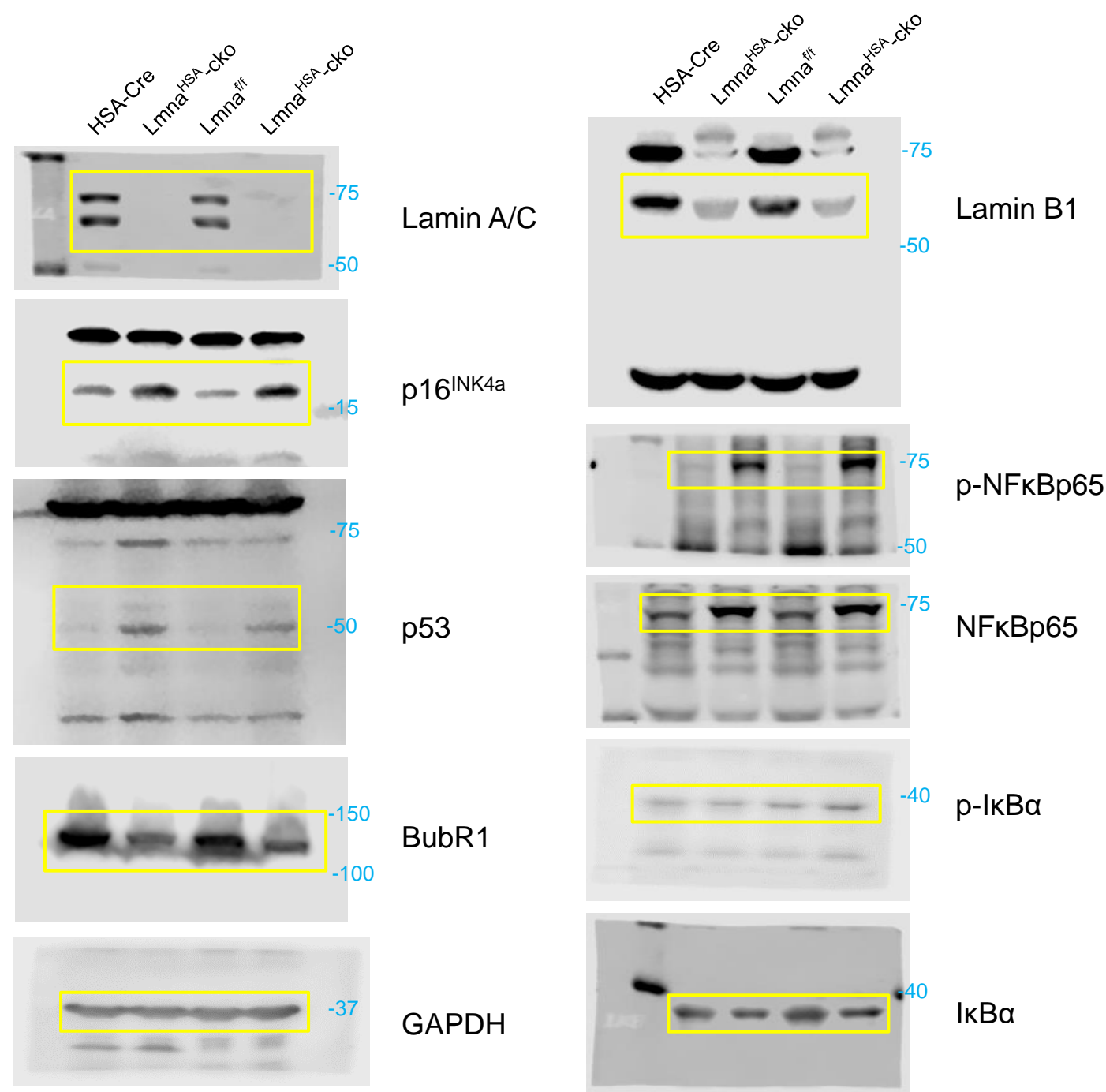

Fig 8F

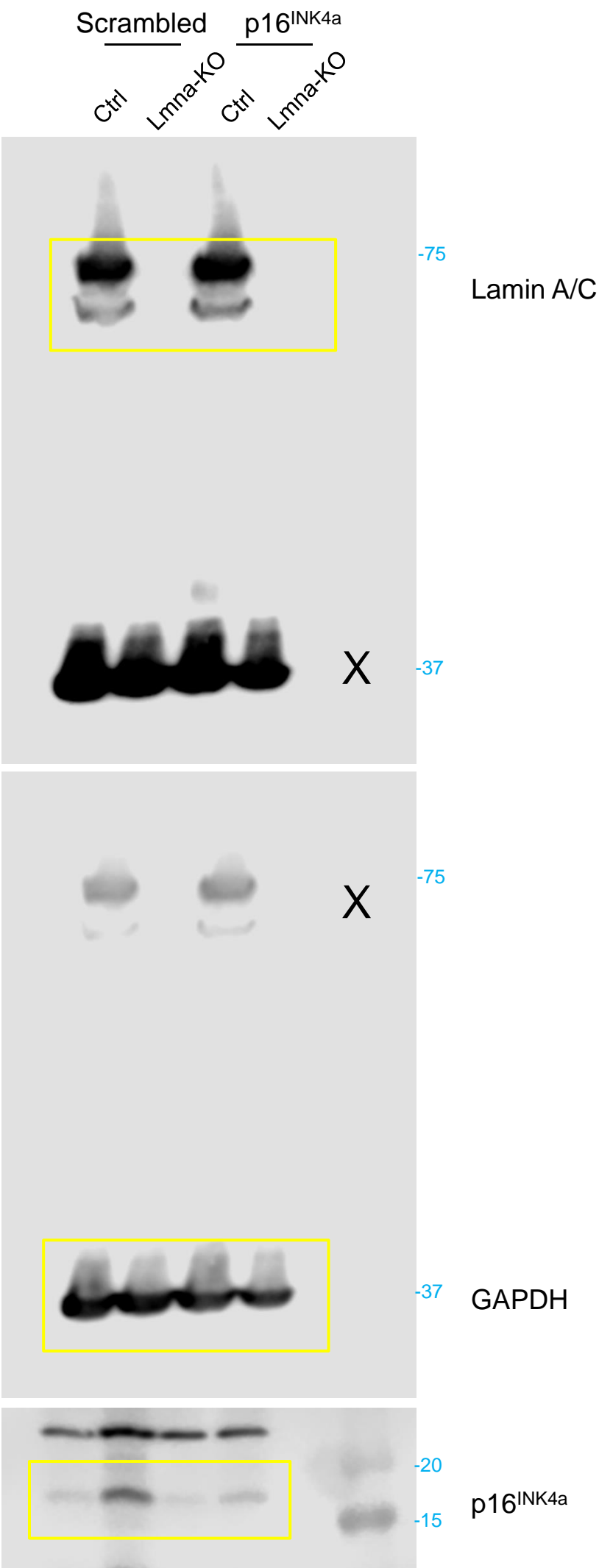

Fig S1B

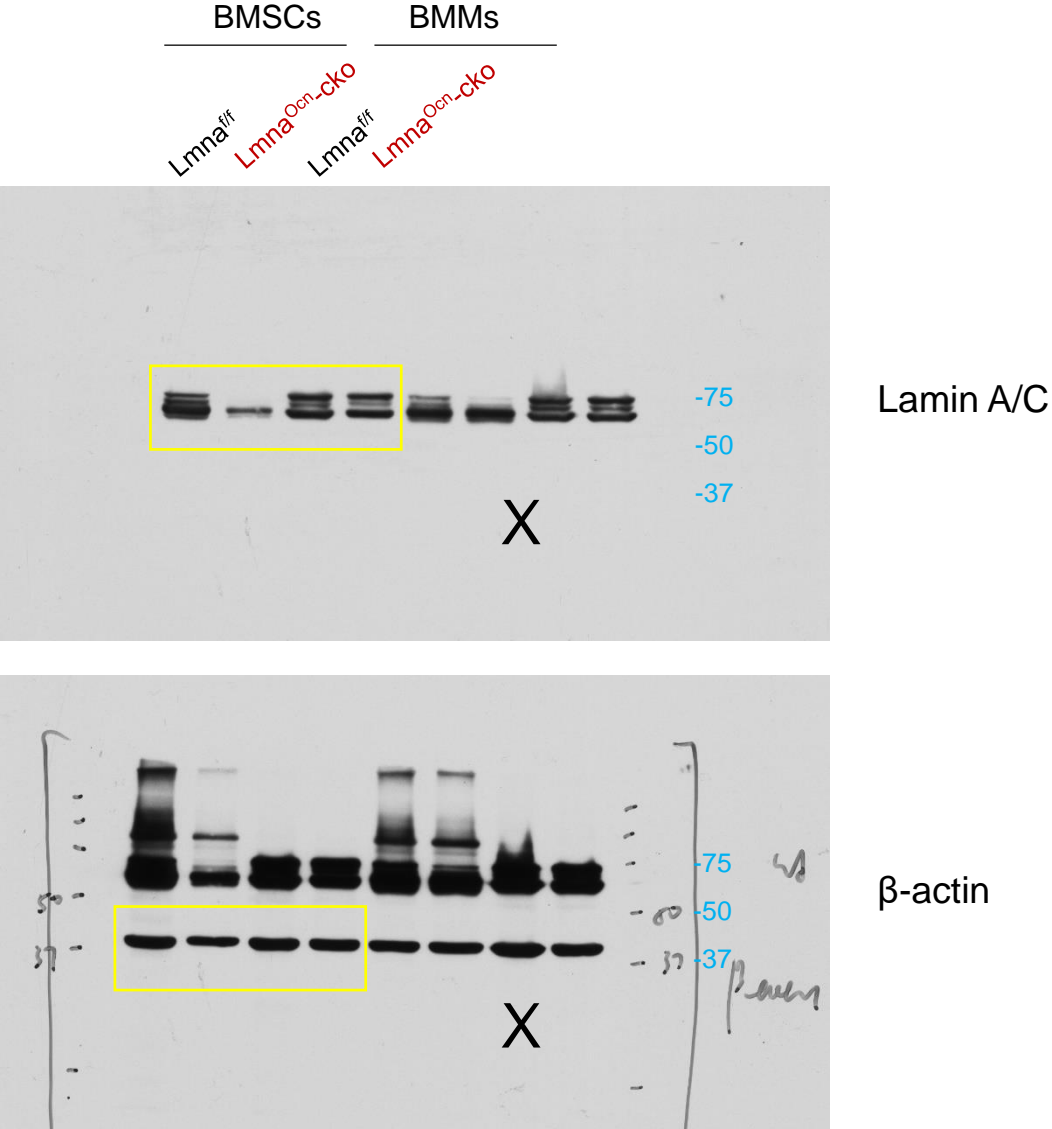

Fig S1C

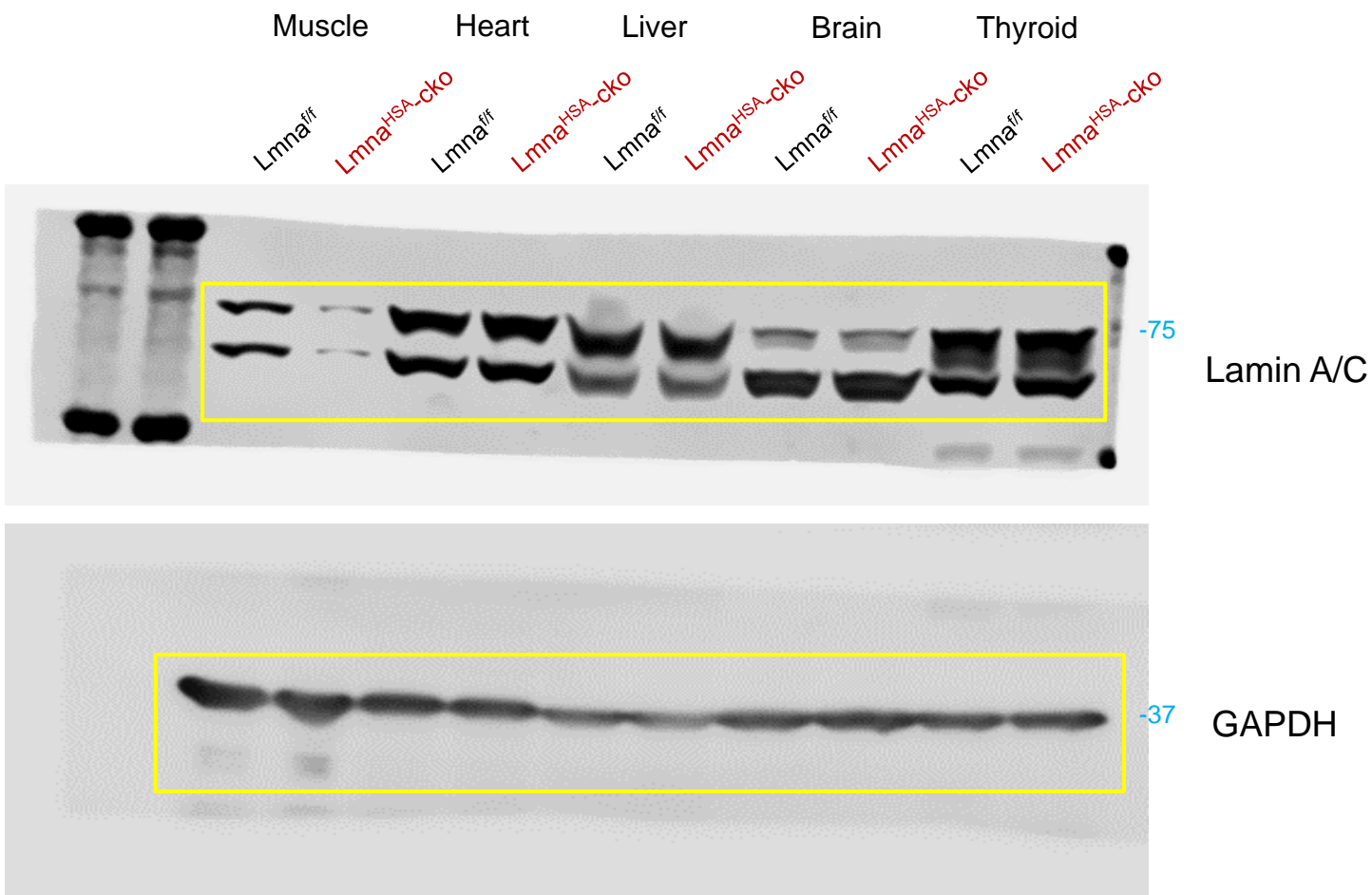

Fig S1D

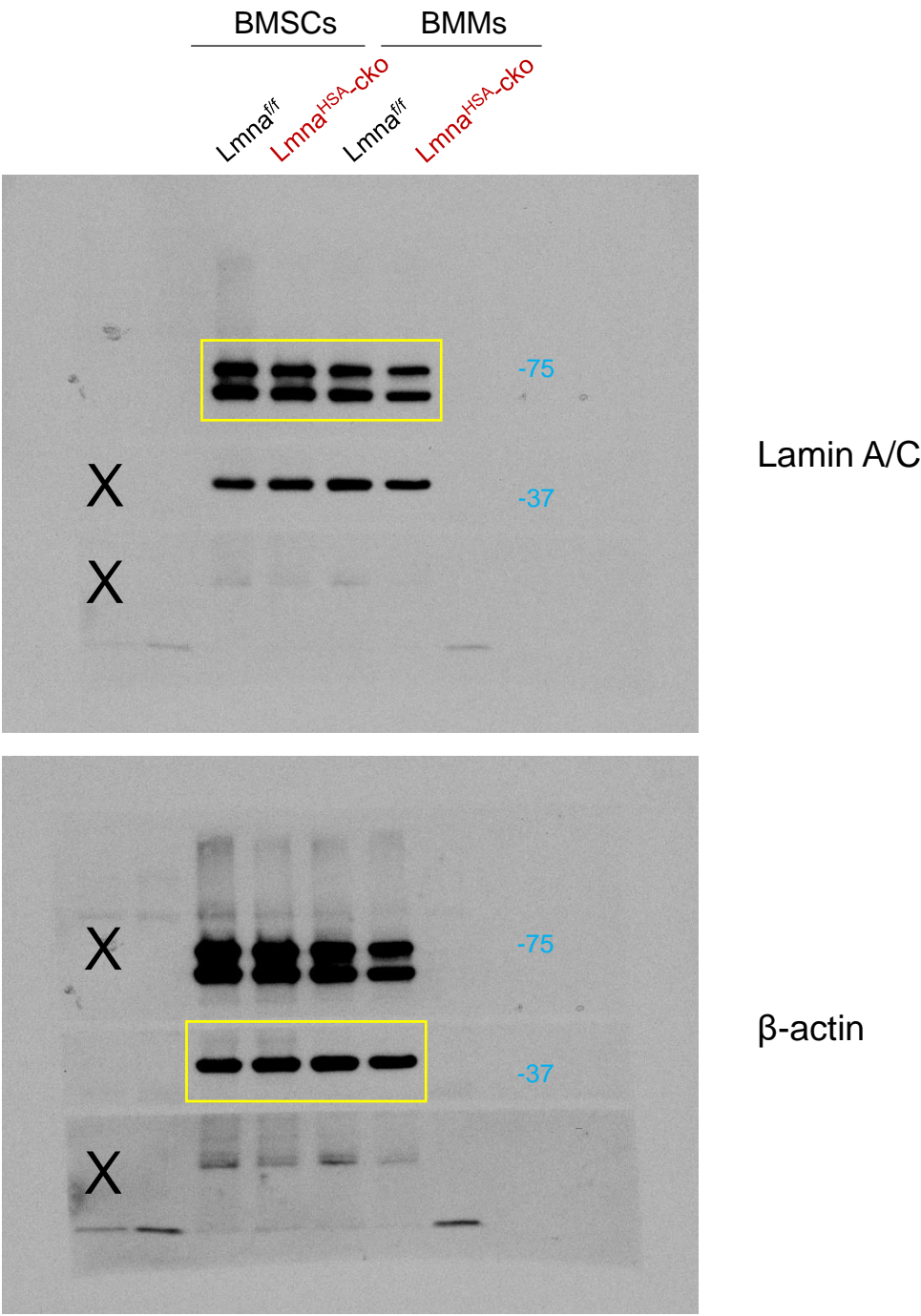

Fig S5B

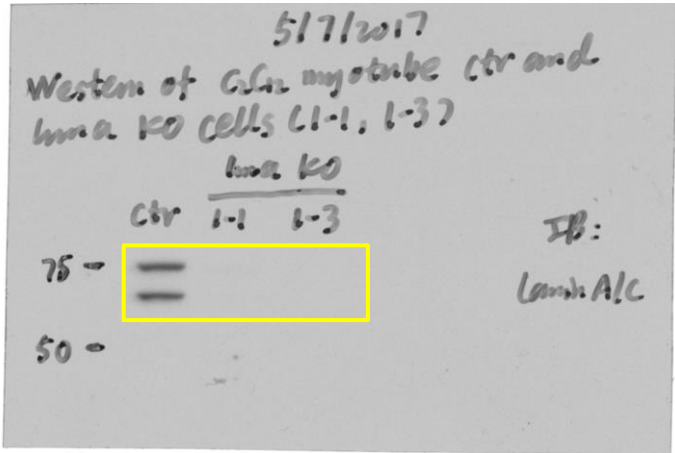

Lamin A/C

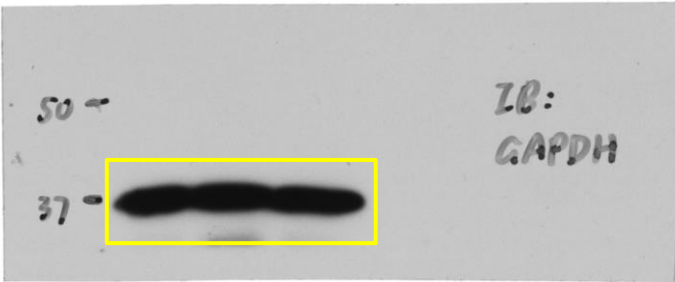

Gapdh

**Fig S10F**

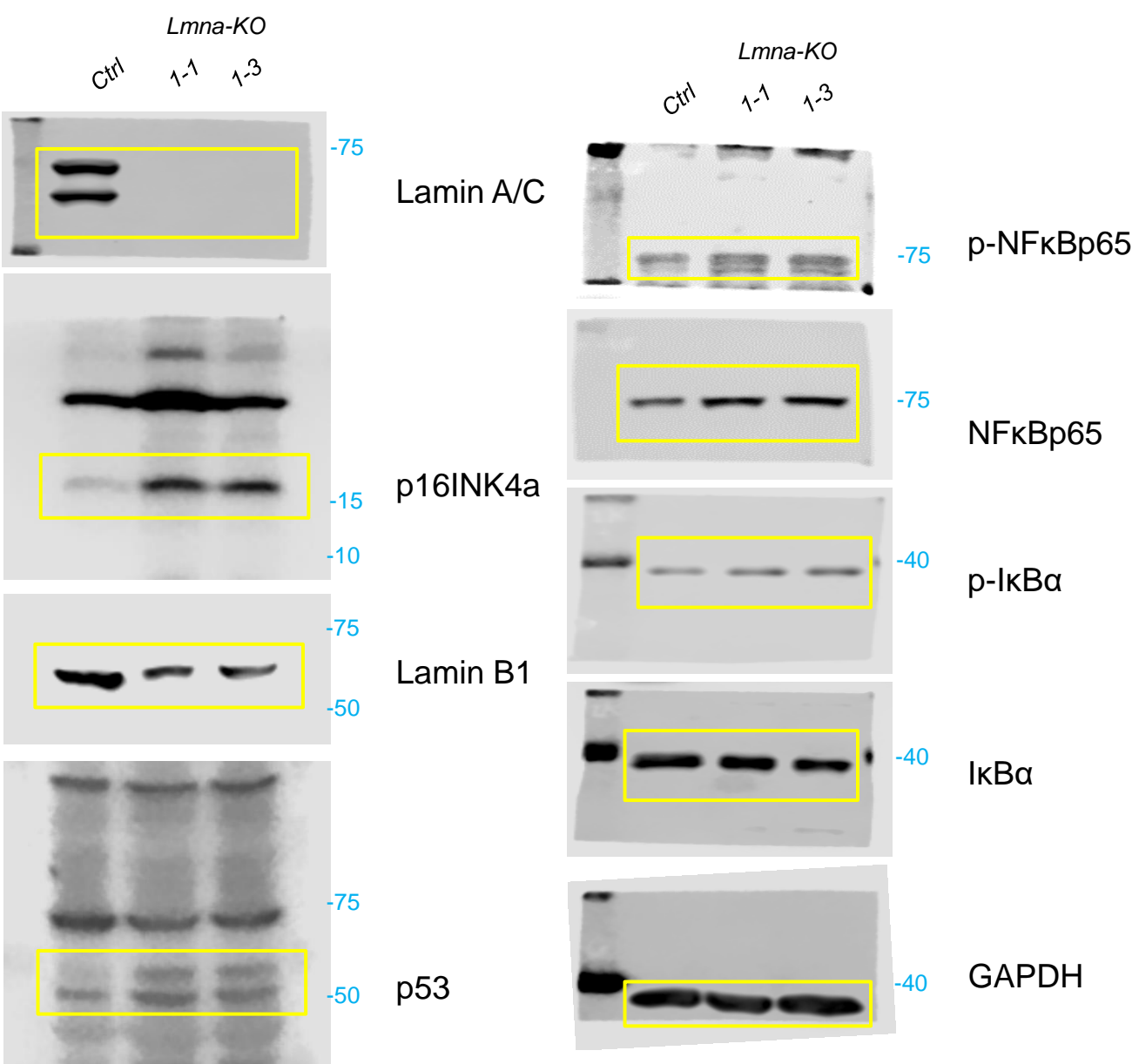

Supplement: S1 Raw Images — The loading order, experimental samples, and molecular weight were indicated. The lanes used in the final figure were marked with a yellow box, and the lanes not used were marked with an “X” above. (PDF) [file pbio.3000731.s012.pdf]
