# Supplementary material for: Linking skeletal muscle aging with osteoporosis by lamin A/C deficiency
Source: PLoS Biol. 2020 Jun 1;18(6):e3000731. doi: 10.1371/journal.pbio.3000731 (PMC7310860; doi:10.1371/journal.pbio.3000731)
Supplement: Attachment — Submitted filename: Response to reviewers.docx [file pbio.3000731.s013.docx]

**Reviewer #1:**

We thank Reviewer 1 for his/her comments that “*L. Xiong et al. provide evidence that skeletal muscle expressed Lamin A/C plays essential roles in preventing not only muscle aging, but also bone loss. The bone loss appears to be due in large to the increase in osteoclast mediated bone resorption. They further carried out mechanistic studies, in particular, antibody array screening for factors secreted from muscle cells, and they identified an increase of IL-6, a pro-inflammatory cytokine, in LaminA/C deficient muscle cells. Moreover, they provide evidence that IL-6 expression is necessary for muscle Lamin A/C-deficiency induced bone loss. Finally, they showed that loss of Lamin A/C in muscles results in senescence and senescence associated secretory phenotype (SASP), which contribute to the increase of IL-6. These are novel findings, and the work provide important insights into muscle Lamin A/C's function, muscle senescence, IL-6, and muscle-bone cross talks. The experiments are well-designed and executed*.”

We also thank the reviewer for his/her constructive suggestions. A point to point response is below.
**1,** “*In Fig S2, 3-month old OB-selective Lmna KO mice showed no change in bone mass. Does Lamin A/C regulate IL-6 expression in osteoblasts? If so, why their bone masses do not change? It is necessary to examine IL-6 levels in the serum and osteoblasts of LmnaOcn-cko mice*.”
**Response:** Good point! We have carried out the suggested experiments. Both IL-6 expression and IL-6 levels in the condition medium (CM) in Lmna-KO and control OB-lineage cells (e.g., BMSCs) were examined. The IL-6 mRNAs and IL-6 protein levels in the CM were increased in Lmna-KO BMSCs, as compared with those of control BMSCs (see revised Fig S7D-S7E). However, the fold of IL-6 increase (~1.7 fold in CM) was less than that in Lmna-KO C2C12 muscle cells (~4 fold) (see revised Figs S7C-S7D). The serum levels of IL-6 in 3-mo control and Lmna^Ocn^-cko mice were also measured, which showed little to no difference in Lmna^Ocn^-cko mice from that of control mice (see revised Fig S7B). These results suggest that whereas Lamin A/C in OB-lineage cells, as that in muscle cells, is necessary to suppress IL-6 expression, its impact on IL-6 expression in OB-lineage cells appeared to be less than that in muscle cells. Thus, the increased IL-6 levels in OB-lineage cells may be in-sufficient to cause bone-loss in 3-MO Lmna^Ocn^-cko mice. However, it is possible that the bone-loss deficit become evident in aged Lmna^Ocn^-cko mice, which we will test in future. These results are described in revised manuscript (see page 11), and these points are discussed in page 19.

**2,** “*The nuclear factor-κB (NF-κB) signaling is a major pathway that stimulates SASP, and the expression of multiple cytokines. Is it altered in Lmna-KO muscles or muscle cells*?”
**Response:** Good suggestion! We have examined NF-kB signaling in control and Lmna-KO muscles and C2C12 muscle cells. Western blot analysis showed increases in both p-NF-κB65 and NF-κB65 levels in Lmna-KO muscles and Lmna-KO muscle cells, as compared with those in controls. In addition, the I-κBα, an inhibitor of the NF-kB65, was decreased in Lmna-KO muscles and C2C12 cells. These data are now included in revised Fig 8C-D and Fig S10F-G, and described in page 14, suggesting an increased NF-κB signaling.

**3,** “*In Fig 8B, in addition to muscle samples, it is necessary to examine p53, another critical transcription factor involved in senescence and cell death, in Lmna-KO muscle cells*.”
**Response:** Agree. The p53 levels were examined by Western blot analysis, which was increased in the mutant muscle cells (see revised Fig S10F, and page 14).

**Reviewer #2:**

We thank Reviewer 2 for his/her comments that “*The manuscript submitted by Lei Xiong et al is an interesting and well designed study aimed at the evaluation of LMNA-linked pathomechanisms causing bone degeneration. The authors take advantage of two different mouse models lacking Lmna expression in skeletal muscle or osteoblasts and show that Lmna-null myoblasts undergo cellular senescence and activated a senescence-associated secretory phenotype featuring high interleukin 6 (IL-6) levels. The authors show that Il-6 secretion determines osteoclastogenesis and bone resorption. Involvement of Il-6 in osteoclastogenesis has been previously proven, thus loss of lamin A/C appears to trigger a known mechanism of bone degeneration, which is selectively inhibited by blocking IL-6. The reported data are extremely interesting from a mechanistic point of view*.”

We also thank the reviewer for his/her concerns/suggestion. A point to point response is below.
**1,** “*loss of LMNA expression is neither the condition of muscular dystrophies nor the condition of progeroid syndromes linked to LMNA. This means that any conclusion should be validated in the presence of a pathogenetic LMNA mutation, a condition more close to human laminopathies.*”

**Response:** Thank the reviewer for raising this point. To address reviewer’s question if loss of LMNA expression occurs in the condition of muscular dystrophies or progeroid syndrome, we examined Lamin A/C levels in muscles of Dmd^mdx^ mice, a mouse model of muscular dystrophy, because the perogeria mouse model is un-available to us currently. Interestingly, Lamin A/C was lower in 3-MO Dmd^mdx^ muscle homogenates. We also measured serum IL-6 levels in Dmd^mdx^ mice (3-MO), which was also elevated, as compared with that of same aged wild type control mice. These results suggest that loss of Lamin A/C expression may occur in the condition of muscular dystrophy. These results were not included in the manuscript, but shown below, because many data (8 Figs, 10 supplemental Figs) have been presented in the manuscript. Given the observations of our manuscript and literature reports that Dmd^mdx^ mice also exhibit reduced bone mass and increased cellular senescence (PMID: 21256750, 26251044, 29883814), these results implicate a contribution of Lamin A/C-loss in Dmd^mdx^ muscles to the IL-6 increase and the bone loss, supporting the view for loss of LMNA expression in muscles as a potential common condition of muscular dystrophies. However, this view requires further examination in future experiments. These points have been included in revised Discussion (see page 18).

(A) Western blotting analysis of Lamin A/C expression in muscles of 3-mo control (WT) and Dmd^mdx^ mice.

(B) Serum levels of IL-6 in 3-mo control and Dmd^mdx^ mice.

**2,** “*although data provided by the authors are convincing, an explanation could be addressed of lack of any effect in osteoblasts upon LMNA downregulation. Moreover, do they undergo cellular senescence? Do they secrete IL-6? Are any other cytokines hypersecreted in Lmna-null osteoblasts*?
**Response:** Thank the reviewer again for raising these suggestions. As responded to Reviewer 1 (point 1), we have examined IL-6 expression and IL-6 protein levels in the CM in Lmna-KO OB-lineage cells (.e.g, BMSCs) and serum IL-6 levels in 3-mo Lmna^Ocn^-cko mice. Although the IL-6 expression was increased in Lmna-KO BMSCs, as compared with that of control BMSCs, the fold of IL-6 increase (~1.7 fold) was less than that in Lmna-KO C2C12 muscle cells (~4 fold) (see revised Figs S7D-S7E), and little to no increase of serum IL-6 level was detected in Lmna^Ocn^-cko mice (see revised Fig S7B), different from that in Lmna^HSA^-cko mice.

To address the question whether Lmna-KO OB-lineage cells undergo cellular senescence, we examined the expression of P16^INK4a^ and P19^ARF,^ both markers of senescence, in Lmna^Ocn-^cko BMSCs. Indeed, both P16^INK4a^ and P19^ARF^ were increased (see Fig S7E). These results suggest that lamin A/C-loss in OB-lineage cells, as that in muscle cells, may undergo cellular senescence and increase IL-6 expression. However, as the impact on IL-6 levels or senescence in OB-lineage cells by Lamin A/C appeared to be less than those in muscle cells, we failed to detect bone-loss phenotype in 3-MO Lmna^Ocn^-cko mice. But, it is possible that the bone-loss deficit become evident in aged Lmna^Ocn^-cko mice, which we will test in future experiments. These results are described in revised manuscript (see page 11), and these points are discussed in page 19.

**3,** -”*In the introduction section there are several conceptual mistakes as well as some typing errors*.”
**Response:** Thanks for point this out. We have made efforts to correct the conceptual mistakes and typo errors (see page 3-5)

**Reviewer #3:**

We thank Reviewer 3 for his/her comments that “*In the manuscript by Xiong et al., the authors set out to understand the mechanism by which loss-of-function in the Lmna gene leads to osteoporosis. To test this, the authors utilizes two different tissue specific Cre-driver mice to delete lamins A and C in either osteoblast-lineage cells or skeletal muscle cells. Somewhat surprisingly, they find that deletion of Lmna specifically in skeletal muscle has a much more significant effect on bone homeostasis compared to deletion in the actual bone. Mechanistically, the authors identify IL-6 as being a molecule that is secreted from Lmna-deficient skeletal muscle cells, which then increases osteoclastogenesis and leads to increased bone resorption. Lastly, the authors propose that the elevated IL-6 is due to senescence of the skeletal muscle cells and the activation of the senescence -associated secretory phenotype. Overall, the authors provide sound evidence that the effect of lamin mutations on osteoporosis may be due to a secretory phenotype, which is potentially independent of loss of contractile forces on the skeletal system. In particular, the timing of the elevated IL-6 prior to any significant muscle dysfunction is consistent with the model that IL-6 is causing the bone resorption. There is clear importance for this work, as it is still not clear how Lmna mutations lead to disease*.”

We also thank the reviewer for his/her concerns/suggestion. A point to point response is below.
**Major Comments
1,** ”*While the authors have presented convincing data the Lmna KO -> p16ink4a -> IL-6 -> osteoclastogenesis is a major contributor impaired bone homeostasis, the data to show that this is due to senescence is very limited, and in my opinion, somewhat speculative. I think that the authors would have to provide significantly more data to conclude that loss of lamins A/C leads to senescence of muscle fibers. One of the hallmarks of senescence is loss of proliferation, which is obviously not relevant in post-mitotic muscle fibers. Furthermore, it seems unlikely that in vitro muscle cells that have been differentiated for only 4-5 days, or the 1-month time point in vivo, is sufficient time to undergo senescence. While p16ink4a is associated with senescence, it can be activated by a number of other things, including ROS and DNA damage, which have also been shown to occur in lamin- deficient or -mutant cells (PMID: 25996284, 16772334, 30696354, 31844279) and these two factors may or may not lead to senescence. If the authors want make a stronger case for the myofibers undergoing senescence, I think they need to provide data for other hallmarks of senescence (i.e. increased β-galactosidase activity, loss of lamin B1), otherwise limit the interpretation to suggest that p16ink4a could be elevated for a number or reasons, including cellular senescence*.”

**Response:** Thank the reviewer for raising this concern. We have examined more markers of senescence, including examining SA-β-galactosidase activity by X-gal staining and lamin B1 levels by Western blot analysis. A marked increase in SA-β-gal activity and a decrease in Lamin B1 levels were detected in Lmna-KO muscles as well as Lmna KO C2C12 cells. These results are included in revised Fig 8 and Fig S10, and described in pages 14 and 28, supporting the view for muscle senescence induced by loss of Lamin A/C. However, as pointed out by the reviewer, while p16^ink4a^ is associated with senescence, it can be activated by a number of other factors, including ROS and DNA damage. In addition, whether the senescence increases IL-6 and promotes bone loss in vivo requires more evidence, which we hope to test it in future experiments. These points have been included in revised discussion (see page 20).

**2,** “*I would be cautious with the nomenclature used to describe the phenotypes. In the laminopathy field, it's fairly well accepted that the progeroid-phenotype is very specific to a subset of mutations. I believe that many would argue that full deletion of the Lmna gene do not reflect the progeroid phenotype (PMID: 24947239). Consider limiting the description of the LmnaHSA-cko mouse to a "laminopathy-like phenotype" to avoid specifically comparing it to the progeria-phenotype*.”

**Response:** Thank the reviewer for the suggestions. Agree, “laminopathy-like phenotype” is a better description. The manuscript has thus been revised.

**3,** “*The authors must clarify what control was used for the Sackcloth mice and include the correct nomenclature in the figures. It is now well recognized that just the expression of Cre can have significant effects in straited muscle (PMID: 29858650, 31125255, 23929940) particularly with respect to DNA damage (PMID: 23929941, PMID: 30917606)*.”
**Response:** Agree! Except for some of the western blot results, most of our previous studies used the litter-mate Lmna^f/f^ as the control, which is now indicated in each legend of figures. As noted by the reviewer, the HSA-Cre may have effects in skeletal muscles (e.g., inducing DNA damage response and increasing expression of pro-inflammatory cytokines), we thus have repeated some of the key experiments by use of both HSA-Cre and Lmna^f/f^ as controls to confirm the phenotypes detected in Lmna^HSA^-cko mice. Indeed, the phenotypes including muscle degeneration (see revised Fig S3), loss of bone mass (see revised Fig S3), IL-6 increases in mutant muscles and serum samples (see revised Fig 5D-E), and increases of senescence markers, gain of SA-β-Gal activity and loss of Lamin B1 (see revised Fig 8A-D) were only detected in Lmna^HSA^-cko mice, supporting previous conclusions.

**Minor Comments**
- “*There are a few instances where I believe laminopathies is misspelt laminopacies*”

**Response:** Sorry for this. The manuscript has been revised.
-”*Description of the expression of lamin levels in Ocn-Cre BMSCs and BMMs in confusion. The way it is written now it seems like the BMMs do not have the transgene, but really it's just not being. Correct?*”

**Response:** Sorry for the confusion. Both BMSCs and BMMs were derived from Lmna^Ocn^-cko mice. The manuscript has been revised (page 6).

-”*It's not completely clear why the HSA-Cre was quantified sometimes after 6 months but the Ocn-Cre was not. Is it possible the Ocn-Cre mice would have developed a phenotype eventually?”*

**Response:** Good point! We used 3-mo mice for most experiments. At this age, Lmna^HSA^-cko mice exhibited obvious phenotypes, whereas Lmna^Ocn^-cko mice show little to no deficit. Agree with the reviewer that Lmna^Ocn^-cko mice may develop phenotypes during aging. As responded to Reviewer 1 (point 1) and Reviewer 2 (point 2), increased IL-6 was also detected in OB-lineage cells from Lmna^Ocn^-cko mice, but at a less degree, as compared with that in mutant muscles or muscle cells. It is possible that the increased IL-6 in OBs is in-sufficient to cause bone loss at 3-mo, but, sufficient to induce deficit upon aging. We hope to test this in future experiments. This point has been included in revised Discussion (see page 19).
-”*The authors need to describe which IL-6 KO strain was used and how the double mutants were generated. There is no mention of this in the methods”*
**Response:** The IL-6 KO mice were purchased from the Jackson Laboratory (Catalog#002650), which are in C57BL/6J background. This information and the generation of the double mutants are now included in revised Materials and Methods (see page 21).

Figures
-*In some cases the figure legends are quite hard to follow, as the description of multiple panels is combined into one sentence. If possible, consider breaking these up to make it easier to follow*.
**Response:** Good suggestion! The figure legends have been revised.

-*Sometimes panel lettering is going across and sometimes is it going down. Again, this just makes the figure hard to follow*.

**Response:** Sorry for this, the figures have been revised.
-*Figure 5C. I believe this is mislabeled with "CM" when the legends states this is skeletal muscle*

**Response:** Yes, should be skeletal muscle.
