# Supplementary material for: Linking skeletal muscle aging with osteoporosis by lamin A/C deficiency
Source: PLoS Biol. 2020 Jun 1;18(6):e3000731. doi: 10.1371/journal.pbio.3000731 (PMC7310860; doi:10.1371/journal.pbio.3000731)
Supplement: Attachment — Submitted filename: Response to reviewers.docx [file pbio.3000731.s014.docx]

**Reviewer #1:**

*The authors addressed all the concerns raised. I would recommend the paper to be accepted for publication.*

**Response:** Thank the reviewer for his/her comments.

**Reviewer #2:**

*The manuscript is a very important research linking with a nice molecular and genetic approach muscle senescence and osteoporosis. Its interest goes beyond the field of lamin-related mechanisms and can provide new hints for osteoporosis and sarcopenia studies. Further, it provides new ideas for the study of laminopathies.*

*Minor changes are needed in grammar and style.*

*Please, add reference to the paper by Rufo et al J Bone Min Res 2011, which is the first study linking muscle disorders to osteoporosis through IL6.*

**Response:** Thank the reviewer for point this out. We have made efforts to revise the writing style, and to correct grammar mistakes in the manuscript and figures (e.g., Fig. S3, ‘Lmna^f/f^’ was mislabeled as ‘Lmnaf^f/f^’, which is corrected). The paper by *Rufo et al J Bone Min Res 2011* is now referenced in revised manuscript (see page 24).

**Reviewer #3:**

*The authors did a very nice job responding to the reviewer’s comments.  These additional experiments greatly enhanced the quality of the manuscript.*

**Response:** Thank the reviewer for his/her comments.
